# Dual rapid lateral flow immunoassay fingerstick wholeblood testing for syphilis and HIV infections is acceptable and accurate, Port-au-Prince, Haiti

**DOI:** 10.1186/s12879-016-1574-3

**Published:** 2016-06-18

**Authors:** Claire C. Bristow, Linda Severe, Jean William Pape, Marjan Javanbakht, Sung-Jae Lee, Warren Scott Comulada, Jeffrey D. Klausner

**Affiliations:** Division of Global Public Health, Department of Medicine, University of California San Diego, La Jolla, CA USA; Les Centres GHESKIO, Port-au-Prince, Haiti; Center for Global Health, Division of Infectious Diseases, Department of Medicine, Weill Cornell Medical College, New York, NY USA; Department of Epidemiology, University of California Los Angeles, Los Angeles, CA USA; Department of Psychiatry and Biobehavioral Sciences, David Geffen School of Medicine, University of California Los Angeles, Los Angeles, CA USA; Department of Medicine, David Geffen School of Medicine, University of California Los Angeles, Los Angeles, CA USA; Division of Global Public Health, University of California San Diego, 9500 Gilman Drive 0507, La Jolla, CA 92093-0507 USA; UCLA David Geffen School of Medicine, Los Angeles, CA USA

**Keywords:** Point-of-care, HIV, Syphilis, Haiti, Dual test, Rapid test, Dual elimination, Haiti, Diagnosis

## Abstract

**Background:**

Dual rapid tests for HIV and syphilis infections allow for detection of HIV infection and syphilis at the point-of-care. Those tests have been evaluated in laboratory settings and show excellent performance but have not been evaluated in the field. We evaluated the field performance of the SD BIOLINE HIV/Syphilis Duo test in Port-au-Prince, Haiti using whole blood fingerprick specimens.

**Methods:**

GHESKIO (Haitian Study Group for Kaposi’s Sarcoma and Opportunistic Infections) clinic attendees 18 years of age or older were invited to participate. Venipuncture blood specimens were used for reference testing with standard commercially available tests for HIV and syphilis in Haiti. The sensitivity and specificity of the Duo test compared to the reference standard were calculated. The exact binomial method was used to determine 95 % confidence intervals (CI).

**Results:**

Of 298 study participants, 237 (79.5 %) were female, of which 49 (20.7 %) were pregnant. For the HIV test component, the sensitivity and specificity were 99.2 % (95 % CI: 95.8 %, 100 %) and 97.0 % (95 % CI: 93.2 %, 99.0 %), respectively; and for the syphilis component were 96.5 % (95 % CI: 91.2 %, 99.0 %) and 90.8 % (95 % CI: 85.7 %, 94.6 %), respectively. In pregnant women, the sensitivity and specificity of the HIV test component were 93.3 % (95 % CI: 68.0 %, 99.8 %) and 94.1 % (95 % CI: 80.3 %, 99.3 %), respectively; and for the syphilis component were 100 % (95 % CI:81.5 %, 100 %) and 96.8 % (95 % CI:83.3 %, 99.9 %), respectively.

**Conclusion:**

The Standard Diagnostics BIOLINE HIV/Syphilis Duo dual test performed well in a field setting in Haiti and should be considered for wider use.

## Background

HIV and syphilis infections cause substantial burden of disease [1–3]. Those with syphilis may be asymptomatic but if left untreated, syphilis can lead to continued transmission, neurological complications, and in pregnant women can result in severe adverse outcomes of pregnancy. Transmission and complications that occur as a result of infection can be prevented through early testing and subsequent treatment for those who test positive.

Up to 80 % of syphilis infections in pregnancy cause adverse outcomes including stillbirths or fetal deaths, neonatal deaths, preterm or low birth weight infants, and infants born with congenital disease [4]. In addition, syphilis infection in HIV infected pregnant women leads to a 2.7 fold increase in the risk of mother-to-child transmission of HIV infection [5]. In order to address the risk of adverse outcomes of pregnancy and the mother-to-child transmission of both HIV and syphilis, the World Health Organization has called for the dual elimination of HIV and syphilis [6].

Integrating the screening of syphilis into HIV prevention programs would add little to the cost of screening but would have a major effect on case finding of both syphilis and HIV and the prevention of transmission [7, 8]. Syphilis and HIV dual testing provides the opportunity to test for both infections using one drop of blood collected with a fingerprick with one device in minutes, allowing for same-day testing and treatment. Dual testing is warranted because both syphilis and HIV infections have evidence-based, scalable interventions using the antenatal care platform for pregnant women [9–11]. Mother-to-child transmission of HIV and syphilis can be prevented through early access to antenatal care, testing and treatment. With the advent of dual testing, HIV and syphilis have affordable and accurate point-of-care integrated tests making it feasible for use in any setting – not just those with laboratory capacity. A dual point-of-care test has the potential to reduce missed opportunities to return results and can improve efficiency along the treatment cascade [11, 12]. The recent development of a new dual rapid HIV and syphilis test is ideal for Haiti, a country with limited resources. In Haiti 90 % of pregnant women report at least one antenatal visit [13]. Therefore, by including rapid syphilis screening in the first antenatal visit, 90 % of pregnant Haitian women could have access to testing and treatment.

The SD BIOLINE HIV/Syphilis Duo test is a qualitative solid phase immunochromatographic assay. It is easy to perform and interpret and does not require special storage or transport conditions with results available in 20 min. The test has been shown to be highly sensitive and specific in laboratory settings using plasma and serum [14–18]. However, rapid tests are intended for use at the point-of-care using a fingerprick whole blood specimen. We evaluated the performance of the SD BIOLINE HIV/Syphilis Duo test in Port-au-Prince, Haiti using whole blood fingerprick specimens.

## Methods

Participants included men and women, at least 18 years of age from GHESKIO (Haitian Study Group for Kaposi’s sarcoma and Opportunistic Infections) clinics in Port-au-Prince, Haiti enrolled from March through July 2014. Working in partnership with the Haitian Government, GHESKIO is a nonprofit organization that provides integrated primary care services, including HIV counseling, AIDS care, antenatal care, and management of tuberculosis and sexually transmitted infections. GHESKIO receives about 100,000 patient visits annually and all of the health care provided by GHESKIO is free of charge, including services and medications. Known pregnant women were actively recruited for participation at the GHESKIO antenatal clinic. Additionally, known HIV and syphilis-infected participants were actively recruited to supplement the study population.

**Fig. 1:**
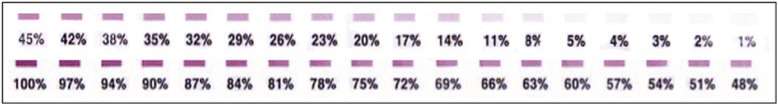
Standard for band color intensity for SD BIOLINE HIV/Syphilis Duo dual rapid test. The color bands on this reference standard were compared to positive results on the Duo test and the percent intensity was recorded by two trained phlebotomists

After participants gave their informed consent, a trained health worker collected a single drop of blood from the participant using a fingerprick. The SD BIOLINE HIV/Syphilis Duo test (Standard Diagnostics, Giheung-gu, Korea) was conducted using the whole blood fingerprick specimen according to the manufacturer’s instructions. In short, the participant’s finger was pricked with a lancet, a capillary pipette was used to collect one drop of blood, the drop of blood was added into the test ‘sample well’ followed by 3 drops of buffer solution. After 20 min the health worker read the test results. The presence of a color band for the control, marked as ‘C’, indicated a valid test. At two other regions marked with ‘SYP’ and ‘HIV’ color bands appeared to indicate positive results. Additionally, the visual intensity of the color band indicating a positive result was recorded by the health worker using the color intensity standard (Fig. 1). The visual color intensity was recorded on a 100-point scale by two separate health workers. We analyzed the average color intensity result between the two.

From the study participants, venipuncture blood specimens were collected by phlebotomists and transported to the reference laboratory for serum separation and reference testing. HIV reference testing varied depending on which clinic at GHESKIO participants presented. The reference tests included the Murex HIV-1.2.0 (DiaSorin S.p.A., Saluggia, Italy) or Alere Determine HIV (Alere Inc., Waltham, MA) rapid tests. Positive results from either test were confirmed with the KHB rapid test (Shanghai Kehua Bio-Engineering Co., LTD, China). For the rapid *Treponema pallidum* antibody reference test, we used the *Treponema Pallidum* Hemagglutination Assay (TPHA) (Human Gesellschaft fur Biochemica und Diagnostica mbH, Wiesbaden, Germany). For TPHA indeterminate results, specimens were retested using a *Treponema pallidum* enzyme-linked immunosorbent assay test (ELISA) (Architect Syphilis TP; Abbott, Wiesbaden, Germany). Rapid plasma reagin (RPR) (Syphilis RPR test, Human Gesellschaft fur Biochemica und Diagnostica mbH, Wiesbaden, Germany) results were also available for all participants to assist with clinical diagnosis. RPR titer levels were determined using serial dilutions.

The sensitivity and specificity of the SD BIOLINE were calculated for the whole study population and for the subset of pregnant women. We also analyzed the results by RPR titer, ≤1:4 and >1:4 because a titer of more than 1:4 is likely to be a recent infection. The exact binomial method was used to determine 95 % confidence intervals (CI) [14]. The Kappa statistic was used to determine concordance between the rapid Duo test and reference test results. Visual color intensity for positive results was summarized using descriptive statistics. All analyses were conducted using SAS v9.3 (Cary, North Carolina, USA). The Institutional Review Board at GHESKIO Centers gave ethical approval for this study.

## Results

Of 298 study participants, 61 (20.5 %) were male. Of 237 females, 49 (20.7 %) were pregnant. The median age of participants was 34 years (interquartile range: 26, 42). All participants had a Duo rapid test and reference tests for antibodies to HIV and syphilis. Of the 298 participants, 21 had inconclusive TPHA results. Of those 21 inconclusive results, all (100 %) were *Treponema pallidum* ELISA positive and 15 (71.4 %) were RPR positive.

The test performance results can be seen in Tables 1 and 2. For the HIV component, the sensitivity and specificity were 99.2 % (95 % CI: 95.8 %, 100 %) and 97.0 % (95 % CI: 93.2 %, 99.0 %), respectively. For the *Treponema pallidum* component, the sensitivity and specificity were 96.5 % (95 % CI: 91.2 %, 99.0 %) and 90.8 % (95 % CI: 85.7 %, 94.6 %), respectively. Two of the 17 false positive results (Duo *Treponema pallidum* positive, TPHA negative) were reactive on the RPR test with titers of 1:4 and 1:64. Among those with higher RPR titers (treponemal antibody positive and RPR > 1:4), the sensitivity for the *Treponema pallidum* component of the test was 97.4 % (95 % CI: 86.5 %, 99.9 %). Additionally, among HIV positive specimens the performance for the *Treponema pallidum* component of the Duo test had a sensitivity of 94.4 % (95 % CI: 72.7 %, 99.9 %) and a specificity of 92.8 % (95 % CI: 86.3 %, 96.8 %) and among HIV negative specimens the sensitivity was 96.8 % (95 % CI: 91.1 %, 99.3 %) and the specificity was 87.8 % (95 % CI: 78.2 %, 94.3 %).

**Table 1:** Field performance of the SD BIOLINE HIV/Syphilis Duo dual rapid test in Haiti for detection of HIV antibodies using a dual HIV/syphilis test, 2014

		HIV reference test*		Total	Sensitivity (95 % CI)	Specificity (95 % CI)	Kappa Coefficient (95 % CI)
		Pos	Neg				
SD BIOLINE **HIV**/Syphilis Duo test	Pos	128	5	133	99.2 % (95.8 %, 100 %)	97.0 % (93.2 %, 99.0 %)	.96
	Neg	1	164	165			(.93, .99)
Total		129	169	298			

*Reference tests were Murex HIV-1.2.0 (DiaSorin S.p.A., Saluggia, Italy) or Alere Determine HIV (Alere) rapid testing. Positive results were confirmed with a colloidal gold test from KHB (Shanghai Kehua Bio-Engineering Co., LTD, China)

**Table 2:** Field performance of the SD BIOLINE HIV/Syphilis Duo dual rapid test in Haiti for detection of *Treponema pallidum* antibodies using a dual HIV/syphilis test, 2014

		*Treponema pallidum* reference test*		Total	Sensitivity (95 % CI)	Specificity (95 % CI)	Kappa Coefficient (95 % CI)
		Pos	Neg				
SD BIOLINE HIV/**Syphilis** Duo test	Pos	109	17	126	96.5 % (91.2 %, 99.0 %)	90.8 % (85.7 %, 94.6 %)	.85
	Neg	4	168	172			(.79, .91)
Total		113	185	298			

*Reference test was conducted using *Treponema Pallidum* Hemaglutination Assay (TPHA) (Human Gesellschaft fur Biochemica und Diagnostica mbH, Wiesbaden, Germany). TPHA indeterminate results were retested using a *Treponema pallidum* enzyme-linked immunosorbent assay test (ELISA)(Architect Syphilis TP; Abbott, Wiesbaden, Germany) and were reclassified based on the ELISA result

In pregnant women, the sensitivity and specificity of the HIV component were 93.3 % (95 % CI: 68.0 %, 99.8 %) and 94.1 % (95 % CI: 80.3 %, 99.3 %), respectively. For the *Treponema pallidum* component the sensitivity and specificity were 100 % (95 % CI: 81.5 %, 100 %) and 96.8 % (83.3 %, 99.9 %), respectively.

The color line indicating a positive result on the Duo test tended to be more intense (darker) for the HIV antibody component (median intensity =100 %) of the positive tests versus the *Treponema pallidum* antibody component (median intensity = 20 %) (Table 3). Additionally, the color band intensity was lower among false positive test results for both HIV and syphilis results than among true positive results.

**Table 3:** Visual intensity of the color of the bands indicating positive results for the SD BIOLINE HIV/Syphilis Duo dual rapid test

	N	Mean (%)	Standard deviation (%)	Median (%)	Quartile range (%)	Minimum (%)	Maximum (%)
HIV band color intensity	133	88.3	28.0	100	0	0.3	100
HIV true positives	128	91.7	22.4	100	0	0.3	100
HIV false positives	5	1.3	2.1	0.3	0.4	0.3	5.0
*T. pallidum* band color intensity*	126	30.3	31.9	20	37	0.3	100
*T. pallidum* true positives	109	34.3	32.4	23	40	0.3	100
*T. pallidum* fase positives	17	4.8	7.1	2	4	0.3	26

*Note: Intensity was not recorded for three of the tests that were syphilis positive on the Duo test

## Discussion

We evaluated a dual rapid point-of-care test for the detection of HIV and syphilis infection in fingerprick whole blood specimens. The HIV antibody component of the dual test showed excellent performance with a sensitivity of 99.2 % and specificity of 97 %. That high performance using whole blood is similar to what was observed in laboratory evaluations using serum or plasma [14–16, 18]. That high performance suggests the dual test could be used at the point-of-care with whole blood fingerprick specimens as a screening test. The few false positive HIV results that were found reinforces the need for HIV confirmatory testing for those who screen positive.

The *Treponema pallidum* antibody component was highly sensitive however the specificity was somewhat lower. False positives could have resulted because the reference test may use different antibody targets than the Duo test, which utilizes Tp0435 (TpN17) recombinant *Treponema pallidum* antigen as the target. Additionally, other reactive antibodies could be responsible for the false positive results observed with the Duo test. We also calculated the performance of the *Treponema pallidum* component of the Duo test stratified by HIV status and found that differences were small with overlapping confidence intervals and therefore are unlikely to be clinically significant.

Among our sample of nearly 300 participants, the Duo gave 17 *Treponema pallidum* false positive results. Of which, two were RPR reactive indicating that these may be falsely negative on the reference test. If we reclassified those as true positives then there would be a slight increase in specificity. That finding highlights the limitations of using an imperfect reference test to determine validity of a new test.

A limitation of all syphilis tests is that they can produce false negative results when the infection has been acquired very recently [19]. Additional limitations occur with treponemal only tests because treponemal antibodies can persist for life, even following curative treatment. Therefore rapid tests for syphilis which detect treponemal antibodies will give a positive result if there is a history of syphilis infection. In some settings confirmatory tests may be necessary while in others – such as antenatal clinics – the benefits of providing same day syphilis treatment might outweigh the risks of unnecessary treatment [20]. A limitation of our study was that we had a modest sample size and used a population of participants from a single city in Haiti. However, because of consistency of the test performance across multiple study populations in various countries, we would not expect those limitations to have a substantial impact [14]. An additional limitation is that for both HIV and syphilis, more than one reference test was used that may not provide the same level of accuracy, although all tests are acceptable validated tests.

Among pregnant women, we found that the *Treponema pallidum* component of the dual test actually had similar or potentially higher sensitivity and specificity compared with the total study population. The risks of untreated syphilis infection in pregnancy are very high and an accurate screening test to detect syphilis is paramount to reduce adverse pregnancy outcomes [4, 6]. The HIV component of the test in pregnant women showed similar and possible somewhat lower performance than among non-pregnant participants, however the sample size was small and the lower performance could be due to random error. Identification of HIV in pregnancy and subsequent intervention is paramount to reduce the burden of mother-to-child HIV transmission.

The color band intensity was subjective. Using an electronic rapid test reader would provide an objective reading of the color band intensity to further explore this relationship [21]. The manufacturer instructions indicate that any color band should be interpreted as a positive result. In our study, false positive results provided a much lower color intensity reading, however due to the substantial overlap in color intensity between false and true positive results, we could not determine an accurate cutoff value.

## Conclusions

The Duo dual rapid syphilis and HIV test is ideal for use in resource-limited settings where laboratory services are often unavailable and follow-up may be difficult. By testing for HIV and syphilis with a single device and one drop of blood from a fingerprick and affordable through bulk procurement at US$1.10, the Duo test has the potential to reduce testing barriers and increase uptake of testing for both HIV and syphilis. The SD BIOLINE Duo test was accurate in this field setting in Haiti and should be considered for expanded use to increase the uptake of dual screening for HIV infection and syphilis.

## Abbreviations

CI, confidence interval; ELISA, enzyme-linked immunosorbent assay; GHESKIO, Haitian Study Group for Kaposi’s sarcoma and Opportunistic Infections; HIV, human immunodeficiency virus; RPR, rapid plasma reagin; TPHA, *Treponema Pallidum* Hemagglutination Assay.
